# A review of initial data on pregnancy during the COVID-19 outbreak: implications for assisted reproductive treatments

**DOI:** 10.5935/1518-0557.20200030

**Published:** 2020

**Authors:** Pedro AA Monteleone, Mayra Nakano, Victor Lazar, Alecsandra P Gomes, Hamilton de Martin, Tatiana CS Bonetti

**Affiliations:** 1 Centro de Reprodução Humana Monteleone; 2 Disciplina de Ginecologia - Departamento de Obstetrícia e Ginecologia. Faculdade de Medicina da Universidade de São Paulo (FMUSP); 3 Departamento de Ginecologia. Universidade Federal de São Paulo - Escola Paulista de Medicina (UNIFESP-EPM)

**Keywords:** COVID-19, SARS-CoV-2, pregnancy, assisted reproduction

## Abstract

The current outbreak of the novel 2019 coronavirus disease (COVID-19) started in China in December 2019 and has since spread to several other countries. On March 25, 2020, a total of 375,498 cases had been confirmed globally with 2,201 cases in Brazil, showing the urgency of reacting to this international public health emergency. While in most cases, mild symptoms are observed, in some cases the infection leads to serious pulmonary disease. As a result, the possible consequences of the COVID-19 outbreak for pregnant women and its potential effects on the management of assisted reproductive treatments, demand attention. In this review, we summarize the latest research progress related to COVID-19 epidemiology and the reported data of pregnant women, and discuss the current evidence of COVID-19 infections during pregnancy and its potential consequences for assisted reproductive treatments. Reported data suggest that symptoms in pregnant women are similar to those in other people, and that there is no evidence for higher maternal or fetal risks. However, considering the initial data and lack of comprehensive knowledge on the pathogenesis of SARS-CoV-2 during pregnancy, human reproduction societies have recommended postponing the embryo transfers and do not initiate new treatment cycles. New evidence must be considered carefully in order to adjust these recommendations accordingly at any time and to guide assisted reproductive treatments.

## Background

The current outbreak of the novel 2019 coronavirus disease (COVID-19) caused by severe acute respiratory syndrome coronavirus 2 (SARS-CoV-2) emerged in China in December 2019 and subsequently spread to many other countries. On January 30 2020, the Emergency Committee of the World Health Organization (WHO) declared a global health emergency, and less than one month later, almost 80,000 cases were confirmed in China and more than 7,000 cases outside of China. Numerous countries have reported increasing numbers of confirmed cases and deaths per day, and despite all efforts, spreading of COVID-19 currently continues; therefore, on March 11 2020, the WHO declared COVID-19 a pandemic (WHO, 2020a).

In numerous countries, current disease dynamics resemble those observed in China following the emergence of COVID-19. On March 5 2020, an issue of Euro Surveillance Journal reported the first confirmed COVID-19 case in Europe (Spiteri *et al* ., 2020), according to the WHO case definition (WHO, 2020b). On March 15 2020, COVID-19 cases have been detected in all 30 countries of the European Union/European Economic Area and in the United Kingdom with a total of 39,768 cases and 1,727 deaths, of which 17,775 cases and 1,441 deaths occurred in Italy alone (European Centre for Disease Prevention and Control, 2020).

The Brazilian Health Ministry confirmed the first COVID-19 case on February 26 2020 in São Paulo. The patient was a 65-year-old man who had recently returned from northern Italy, where a significant outbreak had occurred. This was also the first confirmed case in South America, a continent with a population of over 640 million people who have previously experienced significant outbreaks of infections such as zika, dengue, and measles. The number of COVID-19 cases has been increasing constantly since the first reported case, and while initial cases were associated with travelers arriving from countries with ongoing COVID-19 epidemics, since March 13 2020, the stage of 'community transmission' was announced to be reached in São Paulo and Rio de Janeiro. At the time this manuscript was drafted (March 25 2020), 2,201 cases have been confirmed in Brazil, and the pandemic continues to increase worldwide, with currently 375,498 confirmed cases and 16,362 deaths in 195 countries (WHO, 2020c).

SARS-CoV-2 shows rapid community transmission, and nosocomial infections are common (Novel Coronavirus Pneumonia Emergency Response Epidemiology, 2020). Evidence suggests that when the number of initial cases reaches 40, the likelihood of losing control is high, and the number of infections should double within a week, on average, which highlights the urgency of early detection and rapid response measures (Hellewell *et al*., 2020).

China enacted a program of strict social isolation to control further spreading of COVID-19, with measures including isolation of cases and limited contact, lock-down of cities, mass quarantine, social distancing mandates, school closures, and intense case identification and contact tracing executed by health care professionals (Chen *et al* ., 2020a). These steps helped control local outbreaks, and Chinese authorities reported absence of community transmission cases in China as of March 19 2020. Following this strategy, numerous countries also adopted social isolation regulations to contain the pandemic (WHO, 2020b). However, it is important to emphasize that transmissibility of SARS-CoV-2 is high and infection growth rates are exponential, ranging from 2.2 to 3.6 (Li *et al*., 2020; Zhao *et al*., 2020). The rapid increase in suspected and confirmed cases of COVID-19 suggests that virus transmission may occur by droplet infection and a fecal-oral route. Moreover, transmission from people with mild or no symptoms or before symptom onset may reduce the effect of such isolation strategies (Khan *et al*., 2020; Niud & Xu, 2020; Rothe *et al*., 2020).

## SARS-CoV-2 pathogenesis

Coronaviruses are a large family of viruses known to cause symptoms ranging from a common cold to more severe diseases, such as the severe acute respiratory syndrome (SARS) and the Middle East respiratory syndrome (MERS). The severe acute respiratory syndrome coronavirus (SARS-CoV) caused a SARS outbreak in China in 2002 (Drosten *et al* ., 2003; Ksiazek *et al* ., 2003), and MERS coronavirus (MERS-CoV) was the pathogen responsible for severe respiratory disease outbreaks in the Middle East in 2012 (Zaki *et al* ., 2012). SARS-CoV-2 is the seventh identified member of the family of coronaviruses which infect humans, and the main symptoms including fever, cough and fatigue are similar to those following SARS-CoV and MERS-CoV infection (Liu *et al* ., 2020 a).

Coronaviruses are large, enveloped, positive-sense single-stranded RNA viruses that infect humans and a wide range of animals. SARS-CoV-2 belongs to the genus beta-coronavirus, and it may originate from a virus of bats, as the genome sequence of SARS-CoV-2 is to approximately 90% identical with that of a bat coronavirus. In contrast, SARS-CoV-2 sequences show only about 80% sequence identity with SARS-CoV and about 50% with MERS-CoV (Lu *et al* ., 2020; Zhou *et al* ., 2020).

SARS-CoV-2 has four key structural proteins: the nucleocapsid protein (N), spike protein (S), small membrane protein (SM), and membrane glycoprotein (M). The angiotensin-converting enzyme 2 (ACE2), which is expressed on type-I and type-II alveolar epithelial cells, is the main SARS-CoV-2 receptor, and infection causes respiratory symptoms and eventually the acute respiratory syndrome. This receptor is also expressed in the gut, albeit at a low abundance, and infection may lead to diarrhea and vomiting, despite it is less frequent. The S protein is required for the virus to fuse with the host cell through the receptor-binding domain. This protein includes two subunits, S1 and S2, and while S1 determines cellular tropism, S2 mediates virus-cell membrane fusion. After membrane fusion, viral RNA is released into the cytoplasm, and viral replication is initiated. Newly formed viral particle buds then fuse with the plasma membrane through virion-containing vesicles to release the virus (Guo *et al* ., 2020; Sun *et al* ., 2020). It is noteworthy that SARS-CoV also uses ACE2 as a receptor for cell entry; however, receptor binding ability of SARS-CoV-2 is 10- to 20-fold higher than that of SARS-CoV, and the number of infections with SARS-CoV-2 has exceeded that of SARS infections during the outbreak in China in 2002/2003, indicating higher transmission rates. Moreover, men typically have higher ACE2 levels than women, and Asians show higher levels of ACE2 expression in alveolar cells than Caucasian and African American people, which would suggest Asian males to be most susceptible to infection (Sun *et al* ., 2020).

SARS-CoV-2 is predominantly transmitted from person to person through droplets and close contact (Chan *et al* ., 2020). After contact with a virus-shedding patient, the mean incubation period is about 5 days, ranging from 1 to 14 days (Li *et al* ., 2020). The spectrum of clinical presentations of SARS-CoV-2 infections has been reported to range from asymptomatic infections to severe respiratory failure. However, most cases experience a similar course of disease as SARS and MERS patients, with the most common symptoms including fever and cough which frequently leads to lower respiratory tract disease with poor clinical outcomes in elderly patients and in those with preexisting health conditions. Confirmation of infection requires nucleic acid testing of respiratory tract samples (e.g., pharyngeal swabs), whereas clinical diagnoses can be made based on symptoms, exposure to infection, and chest imaging (Wu & McGoogan, 2020).

The study describing approximately 45.000 cases of COVID-19 in China showed that in most cases (86%), the course of disease was mild (i.e., no or mild pneumonia), and it was severe in 14% (i.e., dyspnea, respiratory frequency ≥ 30/min, blood oxygen saturation ≤ 93%, partial pressure of arterial oxygen to fraction of inspired oxygen ratio <300, and/or lung infiltrates >50% within 24-48 hours) and critical in 5% (i.e., respiratory failure, septic shock, and/or multiple organ dysfunction or failure). The overall case fatality rate was 2.3%, but it was 8.0% in patients aged 70-79 years and 14.8% in patients aged 80 years and older; no deaths occurred in patients aged 9 years or younger and among mild and severe cases. However, case fatality rates were higher in patients with preexisting comorbid conditions, varying from 5.6% to 10.5%, depending on comorbidity (Wu & McGoogan, 2020). SARS and MERS infections, which also showed widespread transmission, produced fatality rates of 9.6% and 35%, respectively (Hui *et al* ., 2020). Despite considerably higher case-fatality rates in SARS and MERS patients, COVID-19 seems to be more transmissible and has led to a higher number of deaths due to the large number of cases. In addition, the true number of COVID-19 cases can be presumed to be higher than the number of reported cases owing to inherent difficulties in identifying mild and asymptomatic cases in addition to insufficient COVID-19 testing capacities in all affected countries (Wu & McGoogan, 2020).

## COVID-19 and Pregnancy

Immunosuppression and other physiological changes during pregnancy cause high susceptibility to respiratory pathogens and severe pneumonia in pregnant women (Jamieson *et al*., 2006) which may require hospitalization in intensive care units and ventilatory support (Goodnight & Soper, 2005). Hormone levels and immune competence show considerable variation throughout pregnancy. Early pregnancy seems to be more risk-prone due to adaptive changes in response to fetal antigens, but conditions typically stabilize with gradual adjustment of the mother's immune and endocrine systems, with highest stability in the late stages of pregnancy. Early pregnancy is a crucial period of fetal organ development, and the immune system is particularly sensitive at this stage, which likely affects the course of infections (Wong *et al*., 2004). Experience with previous respiratory virus epidemics may offer some insights regarding COVID-19 susceptibility and complication rates during pregnancy. Swine-origin influenza A (H1N1) virus is an influenza virus type A, which also causes respiratory disease that can develop into an acute respiratory syndrome. During the H1N1 epidemic in 2009, pregnant women were found to be at higher risk of complications as they were four times more likely to be hospitalized than the rest of the population (Jamieson *et al* ., 2009). Regarding other coronaviruses, the SARS epidemic in 2002/2003 produced 8,442 cases and 916 deaths, and studies have shown that clinical outcomes during this epidemic were worse in pregnant women than in non-pregnant women. In addition, increasing rates of premature births and abortions have been associated with SARS-CoV infections (Schwartz and Graham, 2020). Approximately 50% of pregnant women suffering from SARS required intensive care, and approximately 33% needed mechanical ventilation. The death rate of pregnant women suffering from SARS reached 25% (Wong *et al* ., 2004). From the MERS epidemic which produced 2,500 confirmed cases and caused 858 deaths, we can affirm that MERS progresses much more quickly to respiratory failure and results in higher mortality rates than SARS. However, there was no evidence of vertical transmission of MERS or SARS. Based on this evidence, there is no doubt that SARS-CoV and MERS-CoV infections, as even the H1N1, are associated with higher rates of complications in pregnant women (Schwartz and Graham, 2020).

Despite that COVID-19 epidemic is ongoing and the data are limited, recent reports indicate that clinical characteristics reported in pregnant women with confirmed SARS-CoV-2 infections are similar to those of non-pregnant women with COVID-19 pneumonia, and no evidence of vertical transmission of SARS-CoV-2 in late pregnancy has been produced so far. Nine studies reported COVID-19 in pregnant women, with a total of 69 patients; however, some of these patients may have been included in more than one study (Table 1). The reported cases included five patients in the second trimester of pregnancy, and all other patients were in the third trimester. Most women showed mild or moderate symptoms, and three of them required intensive care. Preterm birth occurred in 8 out of 61 women who gave birth (Chen *et al*., 2020b; Chen *et al*., 2020c; Fan *et al*., 2020; Liu *et al*., 2020b; Liu *et al*., 2020c; Wang *et al*., 2020a; Wen *et al*., 2020; Zhang *et al*., 2020; Zhu *et al*., 2020). A joint investigation carried out by the WHO and China evaluated 147 pregnant women in China (64 confirmed and 82 suspected COVID-19 cases and an asymptomatic patient), 8% showed severe symptoms, and 1% showed a critical course of disease. It was concluded that pregnant women with COVID-19 were not at a higher risk of developing severe symptoms (WHO, 2020d). Most likely, there are many other pregnant women with no or mild symptoms who were not included in these statistics. One case of a neonate infected with SARS-CoV-2 was confirmed 36 hours after birth; however, it is unclear whether this was due to vertical transmission from mother to child (Wang *et al* ., 2020b).

**Table 1:** Characteristic of studies describing pregnant women infected by SARS-CoV-2

Author and year of publication	Local of Study	Nº of pregnant	Age range	Gestational age	Nº of patients at ICU	Nº of deliveries	Nº of preterm births	Vertical transmission
Fan *et a*l., 2020	Hospital of Wuhan University (China)	2	29-34	3rd trimester	0	1	1	none
Chen *et al*., 2020b	Hospital of Wuhan University (China)	9	26-40	3rd trimester	0	9	0	none
Liu *et al*., 2020c	The First Affiliated Hospital of Sun Yat-sen University (China)	13	22-36	2nd trimester (n=2) and 3rd trimester (n=11)	1	10	6	none
Zhu *et al*., 2020	Maternal and Child Health Hospital of Hubei Province (China)	9	25-35	3rd trimester	0	9	0	none
Wang *et al*., 2020a	The Affiliated Infectious Hospital of Soochow University (China)	1	28	3rd trimester	1	1	1	none
Zhang *et al*.2020	Hospital of Wuhan University (China)	16	24-34	3rd trimester	1	16	0	none
Wen *et al*., 2020	Qingdao Women and Children's Hospital (China)	1	31	3rd trimester	0	1	0	none
Liu *et al*., 2020b	Hospital of Wuhan University (China)	15	23-40	2rd trimester (n=3) and 3rd trimester (n=12)	0	11	0	none
Chen *et al*., 2020c	Tongji Medical College (China)	3		3rd trimester	0	3	0	none
Total		69	23-40	2rd trimester (n=5) and 3rd trimester (n=64)	3	61	8	none
Nº: Number; ICU: Intensive Care Unit								

Regardless of the small number of reported cases, the combined data suggest that susceptibility to infection and frequencies of severe courses of disease due to SARS-CoV-2 infection in pregnant women are similar to those in other young adults, and no case of vertical transmission has been reported. Moreover, according to the WHO definition of preterm delivery as birth occurring before 37 weeks of gestation and an estimated rate of preterm births of 10% (WHO, 2018), preterm birth rates in pregnant women affected by COVID-19 seems to follow the general rate. Regarding premature births, it should be considered that many pregnant patients who are hospitalized near term presenting symptoms of COVID-19, the anticipation of delivery by elective cesarean section can be a medical decision that is influenced by the patient and epidemic pressure and is not necessarily a result of current SARS-CoV-2 infection.

No studies on severe COVID-19 and obstetric complications during the first trimester of gestation are available so far; therefore, we lack information on potential effects of infection on pregnancy during the initial stages. Regarding other coronaviruses, SARS and MERS epidemics showed no correlation with frequencies of malformations. Moreover, data from the current epidemic should be considered for managing COVID-19 infections during pregnancy, as the clinical course of this disease and the response to treatments seem to differ from those of previous outbreaks of other types of coronaviruses (Chen *et al* ., 2020d; Liang & Acharya, 2020). Further research is needed in order to understand pathogenesis and epidemiology of SARS-CoV-2 during pregnancy, including aspects such as the time of maternal infection, gestational age, effects of comorbidity factors, and frequencies of adverse outcomes; however, preliminary observations of pregnant women infected with SARS-CoV-2 suggest an optimistic outlook regarding the clinical course.

It is important to consider that the COVID-19 pandemic elicited psychological stress and anxiety in the general population, including pregnant women. Several concerns regarding potential infection during pregnancy have been raised, including (i) presence of family members given quarantine constraints, (ii) potential SARS-CoV-2 exposure during visits to physicians, (iii) potential requirement of early termination of pregnancy through elective cesarean section; (iv) constant use of sodium hypochlorite and alcohol as disinfectants which may exert toxic effects, and (v) potential postpartum complications, e.g., during breastfeeding or neonatal care (Rashidi Fakari & Simbar, 2020).

## COVID-19 and Assisted Reproduction Treatments

In view of the current COVID-19 pandemic and uncertainties regarding effects of SARS-CoV-2 on mothers and fetuses during pregnancy, human reproduction societies published suggestions for managing patients who currently are or will be undergoing infertility treatments through Assisted Reproductive Technologies (ART).

The International Federation for Fertility Societies (IFFS) recommended on March 12 2020 that patients who are considering pregnancy or who are currently undergoing fertility therapies should consult with their personal physician for planning further steps (IFFS, 2020). The same day, the American Society for Reproductive Medicine (ASRM) published a bulletin suggesting that patients who are highly likely to suffer from COVID-19 (i.e., patients who were tested SARS-CoV-2 positive or who have been exposed to confirmed COVID-19 cases within 14 days of onset of their symptoms) should consider freezing oocytes or embryos and avoid embryo transfer until they are symptom-free; however, this recommendation was emphasized to not necessarily apply to suspected COVID-19 cases as symptoms of COVID-19 closely resemble those of other more common forms of respiratory disease (ASRM, 2020a). On March 17 2020, the ASRM published a new document named "Patient Management and Clinical Recommendations During the Coronavirus (COVID-19) Pandemic" in which the key recommendations were: 1. Suspend initiation of new treatment cycles, including ovulation induction, intrauterine inseminations (IUIs), in vitro fertilization (IVF) including retrievals and frozen embryo transfers, as well as non-urgent gamete cryopreservation. 2. Strongly consider cancellation of all embryo transfers whether fresh or frozen. 3. Continue to care for patients who are currently "in-cycle" or who require urgent stimulation and cryopreservation. 4. Suspend elective surgeries and non-urgent diagnostic procedures. 5. Minimize in-person interactions and increase utilization of telehealth (ASRM, 2020b).

The European Society of Human Reproduction and Embryology (ESHRE) issued a statement on March 14 2020 detailing that so far, only few cases of COVID-19 during pregnancy have been reported, thus the respective data must be interpreted with caution as no information is available regarding potential effects of COVID-19 infection during the initial stages of pregnancy; furthermore, medical treatment administered to severe COVID-19 cases may include drugs that are contraindicated during pregnancy (ESHRE, 2020). The same publication advised that all patients considering or planning treatments, independently of confirmation or suspicion of COVID-19 infections, should avoid becoming pregnant at this time and consider deferring pregnancy by freezing oocytes or embryos for embryo transfer at a later point (ESHRE, 2020).

The Brazilian Society for Human Reproduction (SBRH), the Brazilian Society for Assisted Reproduction (SBRA), and the Latin American Network of Assisted Reproduction (REDLARA) also published statements concerning patients undergoing assisted reproductive treatments. The SBRH stressed that there is no cause for panic in pregnant women and urged that the ASRM recommendations for women undergoing or planning infertility treatments be followed. However, treatment plans should be individually discussed with physicians as postponing of treatments may, in some cases, reduce chances of success (SBRH, 2020).

The SBRA and the REDLARA published a joint note on March 17 2020, firstly suggesting that infertility treatments should continue as planned to avoid reducing prospects of success in infertile women but that the advice of international societies to postpone embryo transfers should be followed (SBRA & REDLARA, 2020). Then, an update launched on March 20 2020 recommended that ongoing cycles should be finalized and new procedures should not be initiated. Embryo transfer must be assessed individually with strict controls on the patients and teams involved and the exceptions lies on oncological and others situations in which the postponement may cause loss to the patient, since the decision need to be shared (SBRA & REDLARA, 2020).

It is important to cite the Brazilian Health Ministry published a technical note on COVID-19 and pregnancy on March 25 2020. Based on available evidences until this moment reporting no difference of clinical course, as well as rates of complications and evolution to severe diseases of pregnant compared to young adults; it was recommended that the COVID-19 diagnostic in pregnant women follow the protocol for the general adult population, as the prenatal care for all asymptomatic pregnant women. However, it is highlighted the importance the prevention of agglomerations, best hygiene practices and home screening and isolation of suspected cases of flu syndrome. Also, despite the literature shows the unlikely vertical transmission of SARS-CoV-2, they suggests it is prudent to perform morphological ultrasonography in the second trimester in mothers with SARS-CoV-2 infection, when available, since the data in infected women in the first trimester of pregnancy are not available (BRASIL - Ministério da Saúde, 2020).

Taken together, we currently face a pandemic with a novel virus, and considerable uncertainty remains regarding SAR-CoV-2 infections and consequences of infection during pregnancy. Hence, most of Human Reproduction Societies are restrictive suggesting to postpone embryo transfer independently of confirmation or suspicion of COVID-19 and suspend all cycle's initiations, with rare exceptions. However, whilst every effort must be made to reduce services over coming weeks or maybe months, it is necessary to think forwards towards a resumption of services and a number of questions need to be answered around the management of ART: Should we cancel all ART cycles at this moment? Should we keep the treatments and postpone all embryo transfers? Should we keep only embryo transfers for couple whose success of treatment can be impaired or those who desire transfer the embryos?

## Concluding remarks

Based on most recent epidemiologic data on COVID-19 and pregnancy, there is no evidence to suggest increased risk for mothers or fetuses. It appears that the course of disease after infection with SARS-CoV-2 in pregnant women does not differ from that in other young adults. Moreover, recent evidence suggests no association of vertical transmission and malformations, and the management of pregnant patients should be individualized based on obstetrical indications and maternal/fetal health status. It is important to consider that the current COVID-19 pandemic causes psychological stress and anxiety in pregnant women, which may exert adverse effects. Furthermore, it is important to emphasize the recommendations regarding social isolation and quarantine as issued by health authorities in order to avoid further spreading of SARS-CoV-2. Therefore, deciding between initiating/resuming or postponing assisted reproductive treatments depends more strongly on social isolation than on COVID-19 and its potential effects during pregnancy, bearing in mind potential emotional effects on patients.

However, considering the lack of knowledge regarding SARS-CoV-2 pathogenesis during pregnancy, the current pandemic requires caution and human reproduction societies generally recommended postponing embryo transfers of current cycles and do not initiate any new cycles, with rare exceptions. Nevertheless, we must be alert to new evidence, which can change these recommendations at any time, in order to adjust the management of assisted reproductive treatments.
